# Isothiazologuanosine (^tz^G) as Environmentally Sensitive Fluorescent Reporter in RNA

**DOI:** 10.1002/anie.3552561

**Published:** 2026-09-14

**Authors:** Julia Dietzsch, Yitzhak Tor, Claudia Höbartner

**Affiliations:** ^1^ Institute of Organic Chemistry University of Würzburg Würzburg Germany; ^2^ Department of Chemistry University of California San Diego La Jolla California USA; ^3^ Center for Nanosystems Chemistry (CNC) University of Würzburg, and Cluster of Nucleic Sciences and Technologies – NUCLEATE Würzburg Germany

**Keywords:** fluorescent probes, ribozymes, tRNA

## Abstract

Site‐specific labeling of RNA with emissive, structurally non‐perturbing nucleosides is critically needed for probing RNA structure, dynamics, and function. Isothiazolopyrimidine‐based nucleoside surrogates are leading examples of fluorescent RNA building blocks, with the C‐nucleosidic guanosine analogue ^tz^G standing out due to its brightness. Here, we report the first synthesis of a ^tz^G phosphoramidite and its incorporation into RNA via solid‐phase synthesis. Comprehensive biochemical and photophysical analyses demonstrate that ^tz^G acts as an isomorphic and isofunctional substitute for guanosine, with fluorescence properties that are sensitive to local environmental changes. These features enable fluorescence‐based monitoring of RNA cleavage by deoxyribozymes and of site‐specific RNA methylation by a methyltransferase ribozyme. Our findings establish ^tz^G as a valuable emissive nucleoside analog for the interrogation of RNA modifications at defined positions.

## Graphical abstract

The emissive isothiazologuanosine analog ^tz^G is synthesized for the first time as RNA phosphoramidite building block for solid‐phase synthesis. Due to its isomorphic and isofunctional characteristics, ^tz^G forms Watson–Crick base pairs and can be used as fluorescent probe to monitor (deoxy)ribozyme activity.

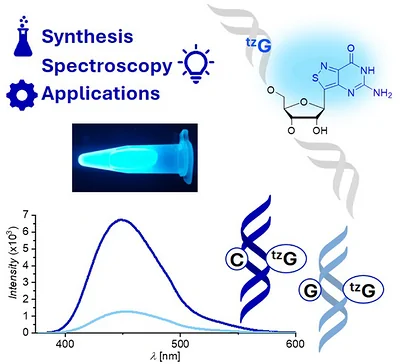

## 1 Introduction

Fluorescence‐based tools are crucial for modern biophysical studies of structure, function, and interplay of biopolymers, as well as small molecule cofactors and messengers [1, 2, 3]. While proteins naturally contain intrinsically fluorescent amino acids, such as tryptophane, canonical RNA and DNA nucleotides are inherently non‐emissive due to fast non‐radiative decay pathways, resulting in short excited‐state lifetimes and negligible fluorescence quantum yields [4, 5]. To overcome this limitation, the development of artificial fluorescent nucleotides has become essential for probing fundamental biochemical processes and enabling the development of biophysical assays. Conventional labeling strategies often rely on bulky exogenous fluorophores that can induce structural perturbations, alter folding dynamics and disrupt functional interactions. Moreover, many existing probes lack environmental sensitivity, limiting their utility for reporting local conformational changes or molecular interactions [6]. Emissive environment‐responsive nucleobase surrogates that mimic the size, structure and function of their native counterparts are needed to overcome these challenges. Alongside 2‐aminopurine (2AP), reported by Stryer in 1969 [7], a large number of base‐pairing, emissive nucleoside analogs including 5‐aryl pyrimidines [8, 9, 10, 11], 8‐furano‐ [9] and 8‐vinyl‐modified purines [12, 13, 14], 6‐aza‐pyrimidines [15, 16], quinazoline derivatives [17, 18] as well as bi‐ [19, 20, 21], tri‐ [22, 23, 24, 25, 26], and tetracyclic [27, 28, 29, 30] nucleobase analogs have been reported. In addition, the emissive RNA alphabet established by the Tor group in 2011 comprises highly fluorescent purines (^th^A, ^th^G) and pyrimidines (^th^C, ^th^U) based on a thieno[3,4‐*d*]pyrimidine heterocyclic core [31].

These isomorphic ^th^N nucleotides have been used for a variety of applications including studies on small molecule cofactors, coenzymes and second messengers [32, 33, 34], monitoring translation [35], PCR labeling of long DNAs [36], transcription [37, 38, 39], structural investigations [40, 41, 42], and fluorescence‐based deamination assays [43, 44]. In the second generation emissive RNA alphabet, the nitrogen atom at the Hoogsteen edge was restored, yielding the isothiazolo[4,3‐*d*]pyrimidine skeleton (Figure 1) [45, 46]. The emissive ^tz^N nucleosides are excellent substrates for metabolic and catabolic enzymes, allowing real‐time monitoring of enzymatic activity by fluorescence [47, 48, 49, 50]. Their microenvironmental responsiveness motivate applications of ^tz^N nucleosides in oligonucleotides, but the synthetic accessibility of ^tz^N‐modified RNA has been severely limiting their widespread use. Until today, the only two reported ^tz^N‐containing RNAs have been enzymatically synthesized using excess of free ^tz^G nucleoside for transcription initiation with a T7 polymerase mutant, followed by enzymatic ligation [51]. However, for the direct site‐specific installation of modified nucleotides, chemical oligonucleotide synthesis is often the method of choice. Here, we report the first synthetic route toward the ^tz^G phosphoramidite and its incorporation into RNA by solid‐phase synthesis, providing convenient access to site‐specifically ^tz^G‐modified RNA. We prepared several hairpin and duplex RNA constructs to explore the response of ^tz^G to different base‐pairing partners in terms of thermal stability and photophysical response. Furthermore, we explored ^tz^G‐modified RNAs as substrates for ribozymes and deoxyribozymes, and found that ^tz^G fluorescence is a useful reporter of DNA‐catalyzed RNA‐cleavage by the 8–17 deoxyribozyme as well as RNA‐catalyzed RNA methylation by the MTR1 ribozyme.

**FIGURE 1:**
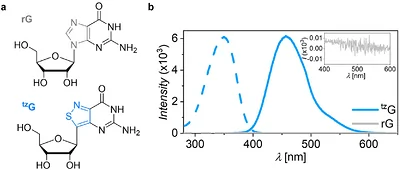
(a) Replacement of the purine scaffold in G with an isothiazolo[4,3‐*d*]pyrimidine heterocyclic core provides the emissive ^tz^G nucleoside. (b) Excitation and emission spectra of rG (grey, insert) and ^tz^G (blue).

## 2 Results and Discussion

The ^tz^G phosphoramidite required for solid‐phase synthesis was prepared via a 16‐step synthetic route including carbon–carbon glycosidic bond formation. First, the free ^tz^G nucleoside was synthesized as reported by the Tor lab [46]. Briefly, synthesis started with acetyl‐ and benzyl‐protection of d‐ribose, followed by Swern oxidation to give the ribonolactone precursor (Scheme S1). This precursor was subjected to alkylation via lithium–halogen exchange and further converted into the free thiol. Thorpe–Ziegler cyclization, stereoselective reduction and guanidinylation prior to deprotection of the exocyclic amine and the sugar hydroxy groups furnished the ^tz^G nucleoside.

Nucleoside incorporation into RNA by solid‐phase synthesis requires orthogonal acid‐, base‐, and fluoride‐labile protecting groups on the nucleobase and ribose. In the first step, a dimethyl formamidine protecting group was installed at the exocyclic amino group of the ^tz^G nucleobase (Scheme 1). This imine group can be cleaved under mildly basic conditions, in contrast to other commonly used nucleobase protection groups (e.g., acetyl and isobutyryl) which require harsher deprotection schemes that could degrade ^tz^G. Next, the base‐protected ^tz^G nucleoside was reacted with 4,4'‐dimethoxytrityl chloride to give the 5'‐DMT‐protected precursor **1**.

**SCHEME 1:**
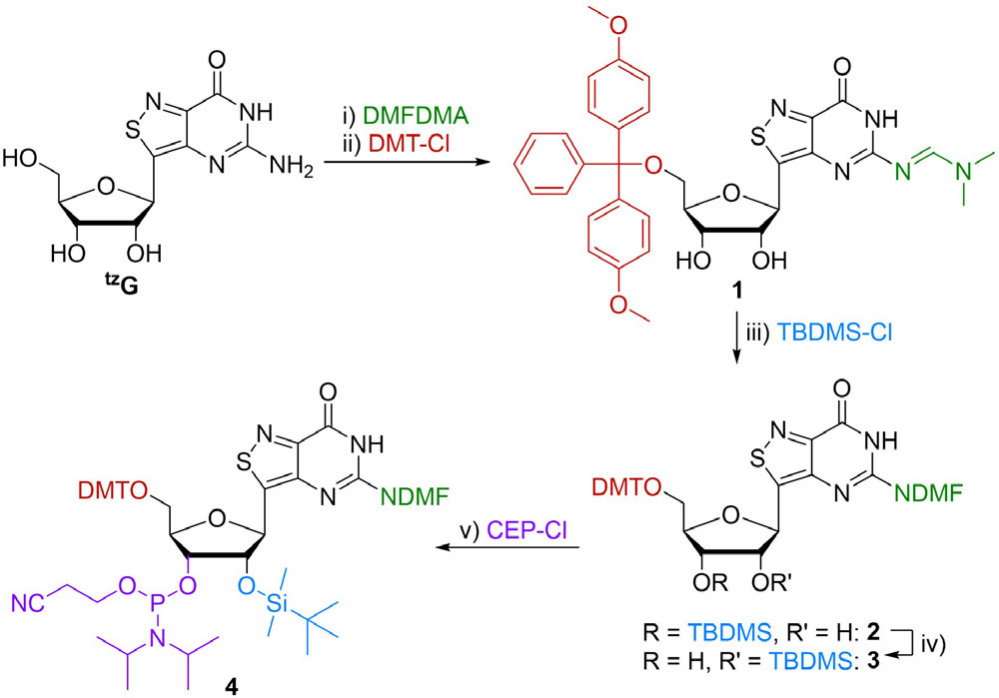
Synthesis of the ^tz^G phosphoramidite building block **4**. Reagents and conditions: (i) Dimethylformamide dimethyl acetal, MeOH, rt, 16 h, 71%. (ii) Dimethylformamide dimethyl acetal, pyridine, rt, 16 h, then DMT‐Cl, pyridine, rt, 16 h, 65%. (iii) TBDMS‐Cl, AgNO_3_, pyridine, rt, 16 h, 60% (2+3). (iv) NEt_3_, MeOH, rt, 30 min. (v) 2‐Cyanoethyl *N*,*N*‐diisopropylchlorphosphoramidit, Hünig's base, DCM, rt, 5 h, 65%.

The most critical step was protection of the 2'‐hydroxyl group. The majority of RNA building blocks used for solid‐phase synthesis are equipped with either a fluoride‐labile TBDMS or TOM group at the 2'‐OH. Upon reaction with the respective silylation reagent both the 2'‐ and the 3'‐protected isomers are formed and need to be separated. Since TBDMS‐protection, in contrast to TOM‐protection, proceeds at ambient temperature and the undesired 3'‐isomer **2** can be converted into the required 2'‐isomer **3** under basic conditions, TBDMS‐ was favored over TOM‐protection. The pure 2'‐protected nucleoside **3** was obtained after several column chromatographic purification steps. Finally, **3** was converted into the phosphoramidite **4** using 2‐cyanoethyl *N*,*N*‐diisopropylchlorophosphor‐amidite in the presence of Hünig's base (see Supporting Information (SI)).

The ^tz^G phosphoramidite **4** was incorporated into the anticodon stemloop of human cytosolic tRNA^Ala(AGC)^ (R1 unmodified, R2 with ^tz^G, Figure 2a) and the 5'‐fragment of mitochondrial tRNA^Trp^ (R9 unmodified, R10 with ^tz^G, Figure S1 and Table S1) by solid‐phase synthesis. Deprotection by overnight incubation in aq. ammonia at 37°C followed by cleavage of the 2'‐silyl groups with TBAF furnished the deprotected RNA, which was purified by PAGE. HPLC chromatograms and HR‐ESI‐MS data for all synthesized RNAs are given in Figures S2, S3, and Table S1.

**FIGURE 2:**
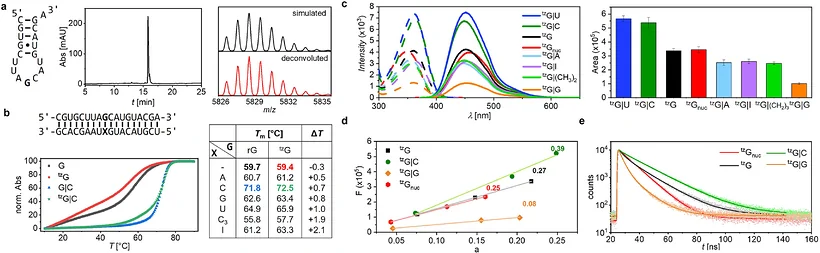
(a) Sequence of the synthesized ^tz^G‐modified RNA (^tz^G shown in bold) with the corresponding anion exchange HPLC chromatogram and the simulated and deconvoluted HR‐ESI mass spectrum. (b) Thermal denaturation analysis for melting temperatures *T*_m_ of 2 µm unmodified (R1) and ^tz^G‐modified hairpin (R2) and duplex samples in PBS with an estimated error of 0.5°C. Δ*T* is the melting temperature difference between unmodified and modified sample. See Table S1 for RNA sequences. (c) Fluorescence excitation and emission spectra for ^tz^G‐containing RNA duplexes (consisting of R2 and R3–R8, see Table S2), the ^tz^G‐modified hairpin R1 and the free ^tz^G nucleoside ^tz^G_nuc_ (left) with the integrated emission intensity for each sample (right). (d) Relative quantum yield analysis with the integrated fluorescence intensity plotted versus the absorption factor a and (e) fluorescence lifetimes for duplexes with ^tz^G opposite C or G in comparison with the ^tz^G nucleoside and ^tz^G in the hairpin. Detailed measurement conditions are given in the Supporting Information.

To evaluate the influence of ^tz^G on hairpin and duplex stability, we compared unmodified (R1) and modified (R2) RNAs as mono‐ or bimolecular constructs upon hybridization with complementary oligonucleotides R3–R8 (containing A, C, G, U, I or acyclic spacer (C_3_) at position X, opposite the G/^tz^G position in R1/R2; Figure 2b). Duplex formation was demonstrated by native PAGE (Figure S4) and thermal stability was analyzed by UV absorbance. The melting curve of the monomolecular hairpin conformation showed that incorporation of ^tz^G into the anticodon loop decreases the melting temperature by only Δ*T*_m_ = –0.3°C compared to the unmodified variant (Figures 2b, S5, and Table S2) [52]. When implemented into 18‐mer duplex structures ^tz^G slightly elevates stability (Δ*T*_m_ = +0.5 to +2.1°C) compared to the unmodified rG‐containing oligonucleotides. The highest thermal stability with *T*_m_ = 72.5°C is observed for the ^tz^G|C base paired duplex. When ^tz^G is placed opposite sterically more demanding purines or the flexible acyclic propyl linker (C_3_), the resulting duplex structures are thermally less stable compared to pyrimidines. Overall, these thermal analyses support the isomorphic character of ^tz^G as it forms a Watson–Crick base pair with C and is well accommodated in both hairpin and duplex RNA structures. These observations are in accordance with reported results for ^th^G‐modified oligonucleotides [31, 40] and predictions by theoretical calculations for ^tz^G [53].

Next, we evaluated the fluorescence of ^tz^G in RNA and its sensitivity toward microenvironmental changes. While many reported fluorescent nucleobase analogs experience quenching after incorporation into oligonucleotides, the emission intensity of the ^tz^G‐modified anticodon stem loop is comparable to that of the free nucleoside (Figure 2c). Upon formation of the ^tz^G|C Watson–Crick and ^tz^G:U wobble base pair, emission is further enhanced by a factor of 1.7. Incorporation of adenosine (A), inosine (I) or a linear alkyl [(CH_2_)_3_] group opposite ^tz^G leads to reduced fluorescence by 50%, likely caused by the increased steric demands of purine nucleobases or the higher flexibility of the acyclic propyl linker. The sterically demanding nucleobase partners can favor non‐radiative decay pathways as crowding promotes excited state distortions and facilitates access to conical intersections, thereby accelerating internal conversion processes and suppressing fluorescence [54]. If ^tz^G is positioned opposite G, its fluorescence is significantly quenched to only 15% of the emission intensity of the brightest ^tz^G:U pairing, likely due to redox‐based quenching mechanisms established for guanine [55]. These observations are also reflected in the emission quantum yield (*Φ*) which increases from 0.25 for the free ^tz^G nucleoside to 0.39 for the ^tz^G|C basepair and is lowest for ^tz^G opposite G with only 0.08 (Figures 2d, S6, and Table S3). These results are in contrast to reported ^th^G‐modified RNA oligonucleotides for which the fluorescence intensity increases with U < A < C < G as opposite nucleobase [31]. This effect might be caused not only by the distinct electronic structures and frontier orbital energies of ^th^G and ^tz^G, but also by structural and environmental effects such as the flanking nucleobases, the sequence context and duplex folding as reported for ^th^G‐containing oligonucleotides (Figure S7 and Table S4) [40, 56]. Fluorescence lifetime analyses by time‐correlated single photon counting (TCSPC) further underline the high responsiveness of ^tz^G to different base pairing partners (Figures 2e and S8). The excited state lifetimes (*τ*) for the ^tz^G nucleoside and ^tz^G in the stemloop are 8.1 and 9.4 ns, respectively, which matches well with a previously determined value for the free nucleoside [57]. Placing ^tz^G opposite C or G in the duplex leads to either a significant increase to 13.3 ns in the case of C or decrease to 4.2 ns for G (Table S3).

These results suggest that ^tz^G‐modified RNA constructs can be used to monitor the catalytic activity of ribozymes and deoxyribozymes by fluorescence spectroscopy. For example, modulating the environment of ^tz^G upon site‐specific RNA‐cleavage or by alkylation of a neighboring nucleoside would be expected to result in detectable photophysical changes.

As a proof of principle for DNAzymes, we chose the 8–17 deoxyribozyme which cleaves RNA site‐specifically between two purine nucleosides [58, 59]. We designed three variants of 8–17 (D1–D3) with the binding arms adjusted for cleavage of the phosphodiester bond 5' of G in R1 and R9, or 3' of G in R9, respectively (Figures 3a and S9). First, we tested if replacement of G by ^tz^G at the cleavage site (i.e., R2 and R10, respectively) is accepted in the DNA‐catalyzed transesterification. All three tested 8–17 DNAzymes produced the expected RNA fragments, demonstrating that ^tz^G is indeed a functional mimic of G (Figures S10–S12 and Table S5). Using Mg^2+^ and Mn^2+^ as cofactors at pH 7.5 or 8.0, the unmodified RNAs were generally cleaved faster than the ^tz^G RNAs (e.g. 1.7‐fold difference in *k*_obs_ (Figure 3b) or larger differences for other buffer conditions (Table S5). Importantly, the fidelity of the cleavage site was maintained, which is essential for fluorescence‐based monitoring of RNA cleavage.

**FIGURE 3:**
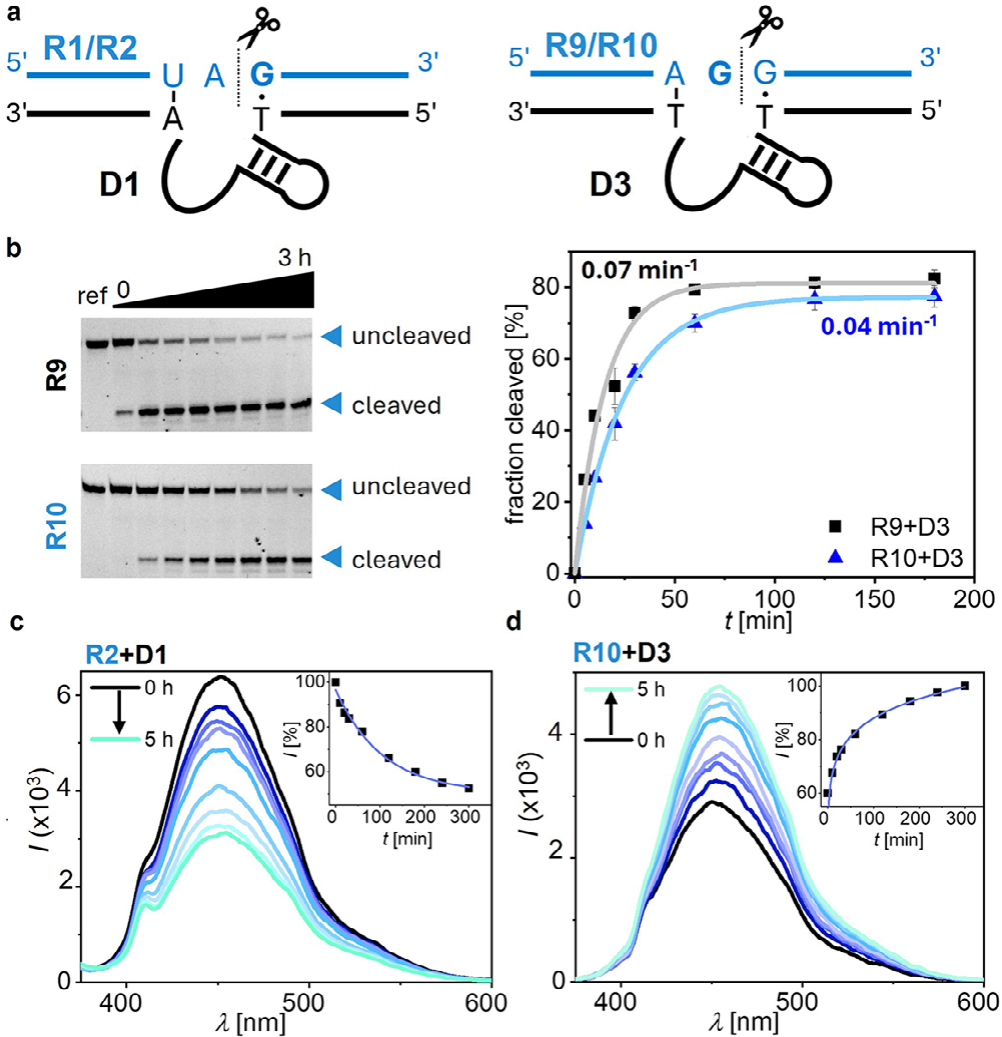
(a) Schematic representation of the cleavage of R1/R2 and R9/R10 by 8–17‐deoxyribozyme variants D1 and D3 with the modified G shown in bold. (b) PAGE analysis and kinetic plot for cleavage of R9/R10 at 25°C. (c, d) Time‐dependent emission spectra and integrated emission intensity (insert) of R2 (c) and R10 (d) at 37°C. Conditions for kinetics: 1 µm RNA, 10 µm DNA, 50 mm Tris‐HCl, 150 mm NaCl, 10 mm MgCl_2_, and 10 mm MnCl_2_.

Indeed, for both R2 and R10, that is, cleavage 3' and 5' of ^tz^G, a time‐dependent change in fluorescence emission was observed upon incubation with the respective 8–17 DNAzymes D1 and D3 and divalent metal ion cofactors (Figure 3c). Surprisingly, D1 resulted in decrease of fluorescence emission for R2, while D3 produced an increase in intensity for R10. These results can be rationalized by changes in the local environment and are consistent with the fluorescence properties described above (Figure 2). For R10, cleavage by D3 leads to separation between ^tz^G and the quenching G residue, thus fluorescence enhancement is observed. On the other hand, in the complex of R2 and D1, ^tz^G is situated in a highly fluorescent ^tz^G:T wobble pair, which is disrupted upon cleavage‐mediated release of the short fragment. We note, however, that alternative explanations exist, since fluorescence is not only affected by the hybridization state and nucleotide environment, but also by buffer conditions and metal ions. Indeed, ^tz^G fluorescence is sensitive to various divalent metal ions (Figure S13). While Mg^2+^ and Ca^2+^ ions barely impact emission, Mn^2+^ causes significant quenching (ca. 50% of the initial intensity), and Co^2+^ and Cu^2+^ cations almost completely abolish emission. In contrast, fluorescence before and after cleavage can be enhanced by denaturation in the presence of 80% formamide (Figure S14). Overall, our data shows that ^tz^G is a suitable fluorescence reporter for the catalytic activity of the 8–17 deoxyribozyme with various RNAs.

Given the environmental sensitivity of ^tz^G's photophysics, we hypothesize that it may be able to monitor postsynthetic RNA modifications at a neighboring position. Building on the promising results seen for DNA‐catalyzed RNA cleavage, we explored the potential of ^tz^G to monitor RNA alkylation by the MTR1 ribozyme, which uses *O* ^6^‐alkylated guanine cofactors and transfers the alkyl moiety to N1 of a defined bulged adenosine (Figure 4a,b) [60, 61].

**FIGURE 4:**
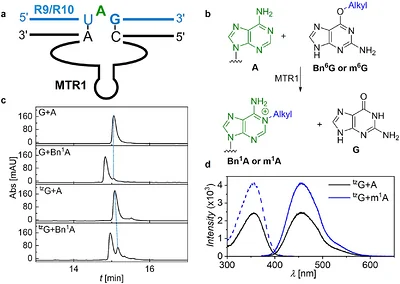
(a) Schematic representation of MTR1 hybridized to R9/R10 substrate RNA. The G position replaced by ^tz^G is shown in bold. (b) MTR1 catalyzes the transfer of an alkyl group from *O* ^6^‐alkylated guanine to N1 of adenosine. (c) Anion exchange HPLC analysis of RNA alkylation with MTR1 and Bn^6^G. 35 µm RNA R9 or R10 were incubated with 45 µm MTR1 and 200 µm Bn^6^G at pH6.0 for 16 h. (d) Excitation and emission spectra for 1 µm R10 in PBS pH7.4, with A or m^1^A next to ^tz^G.

An RNA fragment of human mt‐tRNA^Tyr^, which naturally contains m^1^A at position A9, was chosen (Figure S1). PAGE and HPLC analyses show the unmodified RNA substrates R9 and the ^tz^G‐containing R10 can be both methylated or benzylated using m^6^G or Bn^6^G, respectively, at N1 of the desired adenosine (Figures 4c and S15). Alkylation leads to an increase in ^tz^G emission by up to 70%, either caused by changes in the base‐stacking geometry upon alkylation or reduced photoinduced electron transfer from alkylated adenine to excited ^tz^G (Figures 4d and S15). Therefore, ^tz^G can indeed be used as a fluorescent probe to monitor ribozyme‐mediated RNA alkylation. More broadly, our results demonstrate not only that MTR1 tolerates ^tz^G‐modified RNA as a substrate but also suggest that ^tz^G may be more generally applicable as a sensor for chemoenzymatic RNA modification.

## 3 Conclusion

In conclusion, ^tz^G, an isomorphic and isofunctional G surrogate, has been synthesized and converted into its phosphoramidite and, for the first time, incorporated into RNA by solid‐phase synthesis. Thermal denaturation experiments demonstrate that ^tz^G resembles G in its base pairing behavior while photophysical studies on matched and mismatched ^tz^G‐containing duplexes underline the strong environmental sensitivity of this emissive nucleoside analog. This photophysical feature facilitates fluorescence‐based monitoring of deoxyribozyme‐mediated RNA cleavage and ribozyme‐catalyzed RNA alkylation. This work thus solves the long‐standing challenge of directly accessing site‐specifically modified ^tz^G‐containing RNAs for structural and mechanistic studies of natural or artificial proteins and enzymes or as potential metal ion sensors.

## Author Contributions

**Julia Dietzsch**: investigation, writing – original draft, conceptualization. **Yitzhak Tor**: writing – review and editing, supervision. **Claudia Höbartner**: supervision, writing – review and editing.

## Conflicts of Interest

The authors declare no conflicts of interest.

## Supporting information



The authors have cited additional references within the Supporting Information [62, 63, 64, 65, 66, 67, 68, 69].**Supporting File**: anie74695‐sup‐0001‐SuppMat.docx.

## Data Availability

The data that supports the findings of this study are available in the Supporting information of this article.
