# Supplementary material for: Isothiazologuanosine (tzG) as Environmentally Sensitive Fluorescent Reporter in RNA
Source: Angew Chem Int Ed Engl. 2026 Sep 14;65(43):e3552561. doi: 10.1002/anie.3552561 (PMC13676397; doi:10.1002/anie.3552561)
Supplement: Supplementary file 1 [file ANIE-65-e3552561-s001.docx]

Supporting information for

**Isothiazologuanosine (^tz^G) as Environmentally Sensitive Fluorescent Reporter in RNA**

Julia Dietzsch,*^[a]^ Yitzhak Tor^[b]^ and Claudia Höbartner^[a,c]^

*correspondence: julia.dietzsch@uni-wuerzburg.de

^a^Institute of Organic Chemistry, University of Würzburg, Am Hubland, 97074 Würzburg, Germany

^b^Department of Chemistry, University of California San Diego, 9500 Gilman Drive, La Jolla, CA 92093-0358, USA

^c^Center for Nanosystems Chemistry (CNC), University of Würzburg, and Cluster of Nucleic Sciences and Technologies – NUCLEATE, 97074 Würzburg, Germany

**Table of contents**

[Materials and methods	2](#_Toc220679850)

[Synthesis of tzG phosphoramidite	3](#_Toc220679851)

[Solid-phase RNA synthesis and purification	6](#_Toc220679852)

[3'-Periodate labelling of RNA	7](#_Toc220679853)

[Thermal UV melting curves	7](#_Toc220679854)

[Fluorescence spectroscopy	7](#_Toc220679855)

[Kinetic assays	8](#_Toc220679856)

[Supporting tables	9](#_Toc220679857)

[Supporting figures	12](#_Toc220679858)

[NMR spectra	26](#_Toc220679859)

[References	31](#_Toc220679860)

# Materials and methods

Materials

All standard chemicals including acrylamide/bisacrylamide stock solution, urea and buffer reagents as well as solvents were purchased from commercial suppliers. Organic solvents were used in pro analysis or for synthesis quality without further purification. Solvents for extraction and column chromatography were purchased in technical quality and distilled prior to use. Dry solvents (dichloromethane, THF, DMF, acetonitrile) were obtained from a solvent purification system (SPS). Nanopure water was obtained from a Sartorius Arium® pro ultrapure water system.

Thin layer chromatography (TLC) was performed on silica gel pre-coated aluminum plates (Alugram SIL G/UV254, Macherey-Nagel). Visualization was accomplished by irradiation with UV light at 254 nm. 4,4'-Dimethoxytrityl(DMT)-protected compounds were stained with 5% sulfuric acid in ethanol. Column chromatography was carried out on silica gel (Kieselgel 60, Merck, 0.063–0.200 mm).

The deoxyribozyme sequence was purchased from Microsynth and purified on denaturing PAGE prior to use. RNA oligonucleotides were synthesized in house using solid-phase synthesis with standard phosphoramidite chemistry. 5'-*O*-DMT-2'-*O*-TOM-protected RNA phosphoramidites for solid phase synthesis as well as standard 5'-*O*-DMT-protected CPG supports (1000 Å, 25–35 μmol/g) were purchased from ChemGenes or Sigma Aldrich. SYBR gold and fluorescein-thiosemicarbazide were purchased from Sigma Aldrich. The acyclic propyl linker phosphoramidite was prepared according to a reported procedure.^[1]^

NMR spectroscopy and mass spectrometry

NMR spectra were measured on a Bruker Avance III HD 400 spectrometer at 400 MHz. Chemical shifts (δ) were referenced to the residual solvent signals as internal standards (in ppm; CDCl_3_: ^1^H = 7.26, ^13^C = 77.16, DMSO: ^1^H = 2.50, ^13^C = 39.52) or on the unified scale for ^31^P. Coupling constants (*J*) were reported in Hz. Multiplets were abbreviated as following: s (singlet), d (doublet), t (triplet), q (quartet) and m (multiplet). Spectral assignments were verified by 2D NMR experiments. High resolution ESI mass spectra were measured on a Bruker micrOTOF-Q III spectrometer. Masses for oligonucleotides with m/z below 15,000 are given as the monoisotopic mass, for oligonucleotides with m/z above 15,000 the mass for the peak with the highest intensity is given.

# Synthesis of ^tz^G phosphoramidite

**Scheme S1** Synthetic route towards the ^tz^G phosphoramidite. Compound numbers used in the main manuscript are given in brackets. All reactions that were not including water as a solvent were carried out under nitrogen atmosphere in anhydrous solvents. a) Acetyl chloride, MeOH, rt, 16 h; b) benzyl bromide, tetrabutylammonium iodide, NaH (60% dispersion on oil), DMF, 0 °C to rt, 16 h, 80% over two steps; c) formic acid, H_2_O, 60 °C, 18 h, 70%; d) acetic anhydride, DMSO, rt, 16 h, 70%; e) methyllithium (1.5 m in diethylether), diiodomethane, toluene, -78 °C, 2 h, 61%; f) potassium thioacetate, DMF, 0 °C, 1 h, then rt, 16 h, 66%; g) hydrazine hydrate, DMF, rt, 10 min, then acetic acid, rt, 1 h; h) morpholine, abs. ethanol, 0 °C to rt, 21 h, 72% over two steps; i) triethylsilane, borontrifluoride diethyletherate, DCM, -78 °C, 1 h, then rt, 2 h, 65%; j) ethoxycarbonyl isothiocyanate, acetonitrile, rt, 6 h, then hexamethyldisilazane, 1-ethyl-3-(3-dimethylaminopropyl)carbodiimide hydrochloride, rt, 21 h, 74%; k) 0.25 M NaOH, MeOH, 75 °C, 3 h; l) borontrifluoride diethyletherate, 1,2-ethandithiol, DCM, rt, 3 days, 98% over two steps; m) dimethylformamide dimethylacetal, MeOH, rt, 16 h, 71%; n) dimethylformamide dimethylacetal, pyridine, rt, 16 h, then 4,4'-dimethoxytrityl chloride, pyridine, rt, 5 h, 65%; o) AgNO_3_, tert-butyldimethylsilyl chloride, pyridine, rt, 16 h, 60% (both isomers together); p) diisopropylethylamine, 2-cyanoethyl N,N-diisopropylchlorphosphoramidite, DCM, rt, 5 h, 65%.

The synthesis of the free ^tz^G nucleoside **S14** was conducted according to a published procedure.^[2]^ In the first step, the 1'-OH of ribose (**S1**) was protected with an acetyl group to give compound **S2** which was further converted into the fully protected sugar precursor **S3** by benzyl protection. Cleavage of the acetyl group gave compound **S4** which was oxidized under Swern conditions to the ribonolactone **S5** followed by lithium-halogen exchange to give **S6**. This halogenated intermediate was reacted with potassium thioacetate to thioketone **S7** and reduced to the free thiol **S8**. Thorpe-Ziegler cyclization with tosylated nitrile **S9**^[3]^ yielded compound **S10** which was subjected to stereoselective reduction to give the key intermediate **S10**. Guanidinylation provided the fully protected nucleoside **S12**. Deprotection of the exocyclic amino group gave **S13** which was further converted into the free nucleoside **S14** by removal of the ribose protecting groups. Next, the nucleobase was protected with a DMF group to give **S15**, followed by 5'-DMT protection to **S16**. This intermediate was TBDMS-protected, providing the 3'- and 2'-TBDMS isomers **S17** and **S18**, respectively. The 2'-TBDMS-protected nucleoside **S18** was finally converted into the phosphoramidite **S19**.

DMF-^tz^G (**S15**)

The free ^tz^G nucleoside (**S14**, 500 mg, 1.67 mmol, 1.00 eq.) was dissolved under nitrogen atmosphere in anhydrous methanol (5.5 mL). Dimethylformamide dimethylacetal (0.71 mL, 5.26 mmol, 3.15 eq.) was added and the reaction mixture was stirred at rt for 16 h. After removal of the solvent under reduced pressure, the crude product was purified by column chromatography on silica gel (DCM/MeOH 85:15 to 7:3) to afford compound **S15** (**1**) as a slightly yellow solid (418 mg, 1.18 mmol, 71%).

**^1^H NMR** (400 MHz, DMSO-*d*_6_): *δ* (ppm) = 11.61 (s, 1 H, 1-H), 8.56 (s, 1 H, 10-H), 5.40 (d, *J* = 5.7 Hz, 1 H, 2'-OH), 5.24 (d, *J* = 5.4 Hz, 1 H, 1'-H), 5.03 (d, *J* = 5.3 Hz, 1 H, 3'-OH), 4.93 (t, *J* = 5.6 Hz, 1 H, 5'-OH), 4.17 (q, *J* = 5.1 Hz, 1 H, 2'-H), 3.94 (q, *J* = 4.9 Hz, 1 H, 3'-H), 3.88 (q, *J* = 4.3 Hz, 1 H, 4'-H), 3.66 – 3.56 (m, 1 H, 5'-H), 3.56 – 3.47 (m, 1 H, 5'-H), 3.16 (s, 3 H, 11-H, 11'-H), 3.04 (s, 3 H, 11-H, 11'-H);

**^13^C{^1^H} NMR** (100 MHz, DMSO-*d*_6_): *δ* (ppm) = 158.06 (C-10), 157.10 (C-2), 155.57 (C-6), 150.38 (C-4), 146.05 (C-9), 84.86 (C-4'), 78.05 (C-1'), 76.92 (C-2'), 71.44 (C-3'), 61.86 (C-5'), 34.74 (C-11, C-11'), 34.41 (C-11, C-11');

**HR-MS** (ESI+): *m/z* calc. (C_13_H_18_N_5_O_5_S, [M+H]^+^ ): 356.10232, found: 356.10061.

5'-*O*-DMT-DMF-^tz^G (**S16**, **1**)

Compound **S15** (400 mg, 1.13 mmol, 1.00 eq.) was dissolved in anhydrous pyridine (40 mL) under nitrogen atmosphere. Dimethylformamide dimethylacetal (337 mg, 2.83 mmol, 2.50 eq.) was added and the reaction was stirred at rt for 16 h. The solvent was evaporated and the crude intermediate was redissolved in anhydrous pyridine (40 mL). 4,4'-Dimethoxytrityl chloride (457 mg, 1.35 mmol, 1.20 eq.) was added and the reaction was stirred at rt for 5 h. After removal of the solvent in vacuum and purification by column chromatography on silica gel (DCM/MeOH 98:2), the target compound **S16** (**2**) was obtained as a slightly yellow foam (472 mg, 0.73 mmol, 65%).

**^1^H NMR** (400 MHz, CDCl_3_): *δ* (ppm) = 8.36 (s, 1 H, 10-H), 7.41 – 7.34 (m, 2 H, DMT-H), 7.31 – 7.13 (m, 9 H, DMT-H), 6.84 – 6.75 (m, 4 H, DMT-H), 5.36 (d, *J* = 8.5 Hz, 1 H, 1'-H), 4.41 (dd, *J* = 8.5, 5.2 Hz, 1 H, 2'-H), 4.38 (td, *J* = 3.6, 1.2 Hz, 1 H, 4'-H), 4.35 – 4.30 (m, 1 H, 3'-H), 3.78 (d, *J* = 1.9 Hz, 6 H, DMT-OC*H*_3_), 3.39 (dd, *J* = 10.3, 3.5 Hz, 1 H, 5'-H), 3.22 (s, 3 H, 11-H, 11'-H), 3.16 (dd, *J* = 10.3, 3.8 Hz, 1 H, 5'-H), 3.13 (s, 3 H, 11-H, 11'-H);

**^13^C{^1^H} NMR** (100 MHz, CDCl_3_): *δ* (ppm) = 158.55 (DMT-C), 157.44 (C-10), 157.15 (C-10), 155.20 (C-2), 149.90 (C-5), 149.43 (C-6), 144.74 (C-9), 135.97 (DMT-C), 135.81 (DMT-C), 130.16 (DMT-C), 128.24 (DMT-C), 127.92 (DMT-C), 126.88 (DMT-C), 123.86 (C-4), 113.20 (DMT-C), 86.83 (C-4'), 86.21 (DMT-C), 79.30 (C-1'), 77.36 (C-2'), 73.20 (C-3'), 64.14 (C-5'), 55.32 (DMT-O*C*H_3_), 41.73 (C-11, C-11'), 35.47 (C-11, C-11');

**HR-MS** (ESI+): *m/z* calc. (C_34_H_35_N_5_NaO_7_S, [M+Na]^+^ ): 680.21494, found: 680.21656.

5'-*O*-DMT-2'-*O*-TBDMS-DMF-^tz^G (**S18**, **3**)

Compound **S16** (**1**, 255 mg, 0.40 mmol, 1.00 eq.) was dissolved in dry pyridine (3 mL) under inert atmosphere. Silver nitrate (268 mg, 1.58 mmol, 4.00 eq.) was added and the reaction mixture was stirred for 30 min in the dark. After addition of *tert-*butyldimethylsilylchloride (72 mg, 0.47 mmol, 1.20 eq.) the mixture was stirred at rt for additional 16 h. DCM (20 mL) was added, the mixture was filtered through a Celite pad which was thoroughly washed with DCM, and the solvent was evaporated. Several rounds of column chromatography on silica gel (EtOAc/DCM 99:1 to 9:1) afforded both the 2'- and the 3'-protected isomer (in total 185 mg, 0.24 mmol, 60%). After each column chromatography, the fractions containing the undesired 3'-isomer were collected, the solvent was evaporated and the isomeric mixture was dissolved in MeOH (10 mL) containing triethylamine (1 mL). After stirring at rt for 30 min, the equilibrium with the two isomers in 1:1 ratio was reached, the solvent was removed in vacuum and the mixture was again purified by column chromatography.

**^1^H NMR** (400 MHz, CDCl_3_): *δ* (ppm) = 8.80 (s, 1 H, 1-H), 8.64 (s, 1 H, 10-H), 7.55 – 7.44 (m, 2 H, DMT-H), 7.42 – 7.32 (m, 4 H, DMT-H), 7.33 – 7.24 (m, 2 H, DMT-H), 7.24 – 7.16 (m, 1 H, DMT-H), 6.87 – 6.77 (m, 5 H, DMT-H), 5.51 (d, *J* = 5.5 Hz, 1 H, 1'-H), 4.45 (t, *J* = 5.2 Hz, 1 H, 2'-H), 4.25 – 4.15 (m, 1 H, 4'-H), 4.12 – 4.07 (m, 1 H, 3'-H), 3.78 (s, 6 H, DMT-OC*H*_3_), 3.49 (dd, *J* = 10.4, 2.6 Hz, 1 H, 5'-H), 3.32 – 3.24 (m, 1 H, 5'-H), 3.13 (s, 3 H, 11-H, 11'-H), 3.10 (s, 3 H, 11-H, 11'-H), 2.65 (s, 1 H, 3'-OH), 0.86 (s, 9 H, 14-H, 14'-H, 14''-H), 0.00 (s, 3 H, 12-H, 12'-H), -0.05 (s, 3 H, 12-H, 12'-H);

**^13^C{^1^H} NMR** (100 MHz, CDCl_3_): *δ* (ppm) = 158.62 (C-19), 158.15 (C-19), 157.36 (C-10), 157.27 (C-10), 154.42 (C-2), 150.45 (C5), 146.64 (C9), 144.91 (C4), 136.04 (DMT-C), 135.98 (DMT-C), 130.28 (DMT-C), 130.25 (DMT-C), 128.34 (DMT-C), 128.02 (DMT-C), 126.97 (DMT-C), 113.31 (C-18), 86.45, 83.90 (C-4'), 79.22 (C-2'), 78.28 (C-1'), 72.97 (C-3'), 64.18 (C-5'), 55.37 (DMT-O*C*H_3_), 41.35 (C-11), 35.29 (C-11), 29.84, 25.81 (C-14), -4.53 (C-12), -5.03 (C-12);

**HR-MS** (ESI+): *m/z* calc. (C_40_H_49_N_5_NaO_7_SSi, [M+Na]^+^ ): 794.30142, found: 794.30091.

^tz^G phosphoramidite (**S19**, **4**)

The silyl-protected compound **S18** (**3**, 100 mg, 130 µmol, 1.00 eq.) was dissolved in dry DCM (2 mL) under nitrogen atmosphere. Diisopropylethylamine (181 µL, 1.04 mmol, 8.00 eq.) and 2-cyanoethyl *N*,*N*-diisopropylchlorphosphoramidite (46 mg, 194 µmol, 1.50 eq.) was added and the reaction was stirred at rt for 2.5 h. Since the reaction was not completed, additional 0.7 eq. of 2-cyanoethyl *N*,*N*-diisopropylchlorphosphoramidite were added and it was stirred for another 2 h. After evaporation of the solvent and column chromatography on silica gel (EtOAc/DCM 9:1) the final product **S19** (**4**) was obtained as a white foam (82 mg, 85 µmol, 65%).

**^1^H NMR** (400 MHz, CDCl_3_): *δ* (ppm) = 8.66 – 8.64 (m, 1 H, 10-H), 8.63 – 8.60 (m, 1 H, 1-H), 7.56 – 7.47 (m, 2 H, DMT-H), 7.44 – 7.35 (m, 4 H, DMT-H), 7.32 – 7.23 (m, 2 H, DMT-H), 7.24 – 7.17 (m, 1 H, DMT-H), 6.88 – 6.78 (m, 4 H, DMT-H), 5.57 – 5.52 (m, 1 H, 1'-H), 4.53 – 4.42 (m, 1 H, 2'-H), 4.40 – 4.29 (m, 1 H, 4’-H), 4.17 – 4.05 (m, 1 H, 3'-H), 3.96 – 3.82 (m, 1 H, 17-H), 3.80 – 3.75 (m, 7 H, 17-H, DMT-OC*H*_3_), 3.62 – 3.41 (m, 4 H, 5'-H, 15-H), 3.26 – 3.14 (m, 1 H, 5'-H), 3.14 – 3.07 (m, 6 H, 11-H), 2.77 – 2.53 (m, 2 H, 18-H), 1.34 – 1.16 (m, 6 H, 16-H), 1.14 – 1.08 (m, 6 H, 16-H), 0.85 – 0.82 (m, 9 H, 14-H), 0.01 – -0.03 (m, 3 H, 12-H), -0.04 – -0.11 (m, 3 H, 12-H);

**^13^C{^1^H} NMR** (100 MHz, CDCl_3_): *δ* (ppm) = 158.62 (C-6), 158.21 (C-10), 158.06 (C-10), 157.22 (C-5), 154.36 (C-2), 150.48, 146.62 (C-9), 144.78 (C-4), 135.91 (C-24), 130.29 (DMT-C), 130.24 (DMT-C), 128.46 (DMT-C), 128.36 (DMT-C), 128.04 (DMT-C), 126.97 (DMT-C), 113.32 (DMT-C), 86.56 (DMT-C), 83.02 (C-4'), 78.50 (C-1'), 74.14 (C-3'), 64.21 (C-5'), 58.69 (C-17), 58.68 (C-17), 55.37 (DMT-O*C*H_3_), 42.92 (C-15), 41.36, 35.27 (C-11), 25.93 (C-14), 24.73 (C-14, C-16), 20.36 (C-18), 18.14, -4.53 (C-12), -4.59 (C-12);

**^31^P{^1^H} NMR** (162 MHz, CDCl_3_): *δ* (ppm) = 149.57, 149.04;

**HR-MS** (ESI+): *m/z* calc. (C_49_H_67_N_7_O_8_PSSi, [M+Na]^+^ ): 972.42732, found: 972.43180.

# Solid-phase RNA synthesis and purification

RNA oligonucleotides were prepared by solid-phase synthesis using standard phosphoramidite chemistry with 2'-*O*-TOM-protecting groups on controlled pore glass solid support. Automated solid phase synthesis was carried out on a K&A DNA/RNA synthesizer (H-6/H-8) on a 1 µmol scale. The following solutions were used: 0.25 m ethylthiotetrazole (ETT) in dry acetonitrile as activator, 3% dichloro acetic acid in 1,2-dichloroethane for detritylation, 50 mm iodine in water/pyridine 9/1 for oxidation, pyridine/acetic anhydride/THF 10/10/80 as Cap A solution and 16% NMI in THF as Cap B solution. RNA oligonucleotides were prepared on commercially available CPG solid supports using 70 mm solutions for standard and self-made phosphoramidites.

For unmodified sequences, deprotection was carried out with a mixture of aqueous ammonia and methylamine (1:1) at 37 °C for 4-6 h. The ^tz^G-modified oligonucleotides were deprotected under ultramild conditions by incubation of the solid support in a mixture of H_2_O/methanol/triethylamine (5:4:1) for three days at 25 °C or under harsher conditions in aq. ammonia for 18 h at 37 °C. Deprotection under basic conditions was followed by removal of the 2'-silyl group upon treatment with 500 µL of 1 m tetrabutylammonium fluoride in anhydrous THF at 37 °C for 14-16 h. After addition of 1 m Tris-HCl pH8.0 (500 µL), the THF was removed in vacuum and the deprotected RNA was desalted by size exclusion chromatography using an Äkta start purification system with three HiTRAP Desalting columns (each 5 mL volume). The desalted oligonucleotides were eluted with water and a flow rate of 2 mL/min. The water was removed under reduced pressure and the samples were redissolved in 600 µL of nanopure water.

PAGE purification of the crude oligonucleotides was performed on 300x200x0.7 mm denaturing polyacrylamide gels (20% acrylamide/bisacrylamide 19:1, 7 m urea) with 1x TBE buffer (89 mm Tris, 89 mm boric acid, 2 mm EDTA, pH 8.3). Gels were run at 35 W constant power for 2.5 h. The oligonucleotides were visualized by UV shadowing on a TLC plate. After excision from the gel, the gel pieces were soaked in TEN buffer (10 mm Tris-HCl, 0.1 mm EDTA, 300 mm NaCl, pH 8.0) for oligonucleotide extraction. The pure oligonucleotides were precipitated with absolute ethanol (70% final concentration).

Crude as well as pure oligonucleotides were analyzed by anion exchange HPLC. A Dionex DNAPac PA200 column (2x250 mm) with buffer A (25 mm Tris-HCl, 6 m urea, pH 8.0) and buffer B (25 mm Tris, 0.5 m NaClO_4_, 6 m urea, pH 8.0) was used for this quality control. Samples were run with a gradient of 0-48% B over 12 column volumes with a flow rate of 0.5 mL/min and UV detection at 260 nm. The oven temperature was set to 60 °C. Pure oligonucleotides were additionally analyzed by HR-ESI mass spectrometry (micrOTOF-Q III, negative ion mode, direct injection) and the monoisotopic masses were obtained by deconvolution of the raw spectra. A summary of the oligonucleotides together with their respective measured and calculated molecular masses is provided in Table S1. The anion exchange HPLC chromatograms are summarized in Figure S1.

# In vitro transcription

# In vitro transcription was performed using 100 pmol of template and 100 pmol of T7 promoter. Both were mixed with 5 μL of 10× TxAB annealing buffer and filled up with deionized water to a total volume 50 μL. For annealing, the sample was incubated for 5 min at 95 °C and then cooled to rt for 20 min. Afterwards, 4 μL of 1 m Tris-HCl pH 8.0 (40 mm final concentration), 4 μL of 250 mm dithiothreitol (DTT, 10 mm final concentration), 4 μL of each NTP (100 mm stock, 4 mm final concentration), 3 μL of 1 m MgCl_2_ (30 mm final concentration) and 2 μL of 100 mm spermidine (2 mm final concentration) were added to the annealed mixture and the total volume was brought up to 98 μL using deionized water. After mixing properly, 2 μL of T7 RNA polymerase were added and the sample was incubated overnight at 37 °C. The reaction was stopped by the addition of 80 μL of high dye. The final transcript was purified on 10% denaturing PAGE.

# 3'-Periodate labelling of RNA

3'-End labeling of 3 nmol of RNA was performed by periodate oxidation in 40 µL NaP_i_ buffer (50 mm, pH7.5) mixed with a freshly prepared aqueous solution of NaIO_4_ (20 mm final concentration). After incubation at 37 °C for 10 min, a freshly prepared aqueous Na_2_SO_3_-solution was added to a final concentration of 100 mm followed by incubation for 5 min at 37 °C. After addition of 10 mm fluorescein-5-thiosemicarbazide (100 mm stock in DMSO) the reaction was incubated for another 1 h at 37 °C in the dark followed by purification of the labeled RNA on 20% denaturing PAGE.

# Thermal UV melting curves

Thermal UV melting curves from 2 µm samples in phosphate buffer (100 mm NaCl, 10 mm phosphate, pH7.4) in a final volume of 600 µL were recorded at an Agilent Cary UV–vis Multicell Peltier spectrophotometer. Samples were measured in 10 mm semi-micro quartz cuvettes and overlaid with silicon oil to prevent evaporation during heating. Five temperature ramps with a heating/cooling rate of 0.5 °C/min between 10 and 90 °C at 250, 260 and 280 nm were collected. The melting curves were fitted to a two-state transition model with upper and lower baselines to determine *T*_m_ as previously reported.^[4]^

# Fluorescence spectroscopy

Steady-state fluorescence

Fluorescence spectra were recorded on a Jasco FP-8300 spectrofluorometer equipped with an FCT-817S cell changer and a Julabo F12 temperature control device. 1 µM samples were prepared in 1x phosphate buffer (100 mm NaCl, 10 mm phosphate, pH7.4) and measured in 100 µL Hellma ultra-micro quartz cuvettes (10x2 mm) at 25 °C. Emission spectra were recorded at the excitation maximum (measured emission range *λ*_ex_+15 nm to 700 nm) and excitation spectra were recorded at the emission maximum (measured excitation range 200 to *λ*_em_-15 nm). Excitation and emission bandwidth were both set to 5 nm. Spectra were recorded with 50 ms response, a PMT voltage of 470 V with 0.5 nm data interval and a scan speed of 1,000 nm/min.

Relative quantum yield determination

For quantum yield determination relative to the nucleoside ^tz^G_nuc_, a dilution series (2, 5, 7 μm) of the analyte in 1x phosphate buffer (100 mm NaCl, 10 mm phosphate, pH7.4) was prepared. Fluorescence measurements were conducted in Hellma ultra-micro quartz cuvettes (10x2 mm). Spectra were recorded with excitation at the maximum, emission range λ_ex_+15 to 700 nm, excitation and emission bandwidths 2.5 nm, scan speed 1,000 nm/min, data interval 0.5 nm, response time 50 msec, PMT voltage 520 V. After fluorescence measurement, absorption spectra were recorded in the same cuvettes.

Fluorescence lifetime measurements

Fluorescence lifetimes were determined by TCSPC using a Horiba DeltaFlex spectrometer with a DeltaDiode excitation source (DD-405L λ_ex_ 318 nm) under magic angle conditions (54.7°) of polarization filters. 1 μm samples in 1x phosphate buffer (100 mm NaCl, 10 mm phosphate, pH7.4) were measured in Hellma ultra-micro quartz cuvettes (10×2 mm). The emission bandwidth was adjusted for a stop rate of close to 2%. The photon arrival time was set to 4096 channels (0.051 ns per channel), and data was collected until 10,000 counts had accumulated in the peak channel. The instrument response function was recorded from only buffer by setting the emission monochromator to the peak wavelength of the excitation source. Fluorescence decay curves were analyzed by iterative reconvolution fitting with the instrument response function using the algorithm implemented in Horiba DAS6 (version 6.8).

Cuvette-based kinetic assay

Cuvette-based kinetics were carried out on a Jasco FP-8300 spectrofluorometer equipped with an FCT-817S cell changer and a Julabo F12 temperature control device. Samples were measured in Hellma ultra-micro quartz cuvettes (1.5x1.5 mm) and contained 1 µm RNA and 10 µm DNA in a final volume of 20 µL. Prior to measurement, the required amount of DNA and RNA was diluted with water to a volume of 16 µL, annealed for 4 min at 95 °C and cooled to rt over 10 min. Afterwards, 2 µL of 10x kinetic reaction buffer (500 mm Tris-HCl, 1.5 m NaCl, pH7.5 or pH8.0) and 200 mm MgCl_2_ and 200 mm MnCl_2_ solution were added to either 10 or 20 mm final concentration. Samples were incubated in cuvettes at 37 °C and excitation and emission spectra were recorded at 0 min, 10 min, 30 min, 1 h, 2 h, 3 h, 4 h, 5 h, 6 h and 21 h.

# Kinetic assays

Kinetics characterization was carried out under single turnover conditions. The deoxyribozyme (100 pmol) was mixed with the fluorescently labeled RNA substrate (10 pmol) in a final volume of 8 μL. The resulting mixture was heated to 95 °C for 4 min and then cooled to 25 °C for 10 min. To initiate the reaction, 10x kinetic assay buffer (1 μL, 500 mm Tris-HCl, 1.5 m NaCl, pH7.5 or 8.0) and MgCl_2_ (1 μL, 200 mm) or MgCl_2_ and MnCl_2_ (1 µL, 200 mm) were added and the resulting mixture was incubated at 25 or 37 °C. Aliquots (1 μL) were taken at different time points, quenched with loading buffer and analyzed by denaturing PAGE. Cleavage yields were determined by measuring the fluorescent intensities of the corresponding bands. *k*_obs_ values (observed cleavage rate) were obtained by fitting fraction cleaved versus time with the first-order kinetics equation Y = Y_max_ * (1 − e^−^*^k^*^obs*^*^t^*). Each experiment was repeated at least three times, and values are reported as mean of three individual experiments.

For analysis by reverse phase HPLC, samples were diluted to a final volume of 110 µL and a final concentration of 100 mm TEAA pH7.0. Samples were injected on a JASCO HPLC system onto a XBridge C18 column from Waters with buffer A (100 mm TEAA pH7.0) and buffer B (100 mm TEAA pH7.0 in 25% MeCN) and run at 60 °C with a gradient of 15 to 60% buffer B over 26 minutes and a flow of 1 mL/min. Chromatograms were recorded at an absorption wavelength of 260 nm and a fluorescence emission wavelength of 349 nm.

# Alkylation by MTR1

For analysis of MTR1 alkylation on PAGE, 100 pmol of MTR1 and 10 pmol of fluorescently labelled RNA substrate were dissolved in 8.6 µL of buffer (50 mm BisTris, 120 mm KCl, 5 mm NaCl, pH6.0). After annealing for 3 min at 95 °C and cooling to rt, m^6^G or Bn^6^G (0.4 µL, 200 µm final concentration) and MgCl_2_ (1 µL, 40 mm final concentration) were added and the reaction mixture was incubated at 25 °C. 1 µL aliquots were taken at different time points, quenched with loading buffer and analyzed by 20% denaturing PAGE.

For analysis by HPLC and fluorescence, 175 pmol of unlabelled RNA substrate and 225 pmol of MTR1 were dissolved in 4.7 µL of buffer (50 mm BisTris, 120 mm KCl, 5 mm NaCl, pH6.0), annealed for 3 min at 95 °C and cooled to rt. 0.1 µL of 10 mm m^6^G or Bn^6^G (final concentration 200 µm) and 0.2 µL of 1 m MgC_l2_ (final concentration 40 mm) were added and the reaction was incubated overnight at 25 °C. After addition of 1 µL 0.5 m EDTA, the sample was split in half for analysis by HPLC or fluorescence.

The m^1^A-containing substrate was isolated by anion exchange HPLC using a Dionex DNAPac PA200 column (4x250 mm) with buffer A (25 mm Tris-HCl, 20% MeCN, pH 8.0) and buffer B (25 mm Tris, 0.5 m NaClO_4_, 20% MeCN, pH 8.0). Samples were run with a gradient of 0-48% B over 12 column volumes with a flow rate of 1 mL/min and UV detection at 260 nm. The oven temperature was set to 60 °C. Collected peaks were lyophilized and redissolved in water. A 1 µm sample in PBS (100 mm NaCl, 10 mm phosphate, pH7.4) was prepared for fluorescence measurements.

**Theoretical calculations**

Quantum chemical calculations were performed with ORCA Version 6.1.1.^[5-6]^ Ground-state geometries were optimized using density functional theory (DFT) at the ωB97X-D3/def2-TZVP level of theory.^[7-9]^ Frequency calculations at the same level confirmed all optimized structures as true minima on the potential energy surface by the absence of imaginary frequencies. HOMO and LUMO energies were obtained from the converged Kohn–Sham wavefunctions at the ωB97X-D3/def2-TZVP level of theory in the gas phase.

# Supporting tables

**Table S1:** RNA oligonucleotides prepared by solid-phase synthesis and ordered DNA oligonucleotide together with calculated and measured HR-ESI-MS masses.

| **No.** | **Description** | **5'-sequence-3'** | **Calculated mass** | **Found mass** |
| --- | --- | --- | --- | --- |
| R1 | unmodified hairpin (18 nt) | pCGUGCUUA**G**CAUGUACGA | 5809.75 Da | 5809.76 Da |
| R2 | ^tz^G-modified hairpin (18 nt) | pCGUGCUUA**^tz^G**CAUGUACGA | 5826.71 Da | 5826.77 Da |
| R3 | counterstrand ^tz^G\|A (18 nt) | UCGUACAUG**A**UAAGCACG | 5736.82 Da | 5736.84 Da |
| R4 | counterstrand ^tz^G\|C (18 nt) | UCGUACAUG**C**UAAGCACG | 5712.80 Da | 5712.90 Da |
| R5 | counterstrand ^tz^G\|G (18 nt) | UCGUACAUG**G**UAAGCACG | 5752.81 Da | 5752.82 Da |
| R6 | counterstrand ^tz^G\|U (18 nt) | UCGUACAUG**U**UAAGCACG | 5713.79 Da | 5713.80 Da |
| R7 | counterstrand ^tz^G\|I (18 nt) | UCGUACAUG**I**UAAGCACG | 5737.80 Da | 5737.89 Da |
| R8 | counterstrand ^tz^G\|C_3_ (18 nt) | UCGUACAUG**(CH_2_)_3_**UAAGCACG | 5545.77 Da | 5545.78 Da |
| R9 | unmodified tRNA fragment (20 nt) | AGAAAUUUA**G**GUUAAAUACA | 6404.91 Da | 6404.94 Da |
| R10 | ^tz^G-modified tRNA fragment (20 nt) | AGAAAUUUA**^tz^G**GUUAAAUACA | 6421.87 Da | 6421.75 Da |
| R10-m^1^A | MTR1-methylated R10 | AGAAAUUUm^1^A**^tz^G**GUUAAAUACA | 6435.88 Da | 6435.91 Da |
| D1 | 8-17 deoxyribozyme for A\|**G** in R1/R2 (31 nt) | TCGTACATGTGTCAGCGACTCGAAAAGCACG | 9524.63 Da | 9524.69 Da |
| D2 | 8-17 deoxyribozyme for A\|**G** in R9/R10 (44 nt) | GGCTCTTGGTCTGTATTTAACTGTCAGCGACTCGAAAAATTTCT | 13496.25 Da | 13496.19 Da |
| D3 | 8-17 deoxyribozyme for G\|**G** in R9/R10 (44 nt) | GGCTCTTGGTCTGTATTTAATGTCAGCGACTCGAATAAATTTCT | 13511.25 Da | 13511.19 Da |
| MTR1 | MTR1 for A9 alkylation of R9/R10 (59 nt) | GGUAUUUAACCUGACCGACCCCCCGAGUUCGCUCGGGGACAACUAGACAUAAAAUUUCU | 19061.43 Da | 19061.72 Da |

**Table S2:** Melting temperatures *T*_m_ for 2 µm hairpin and duplex structures. Given are also the mean *T*_m_ and standard deviation (SD) of four ramps as well as the melting temperature difference compared to the unmodified hairpin or Watson-Crick base-paired duplex Δ*T*_m_.

| **No.** | **5'-NNN-3'**  **3'-NNN-5'** | ***T_m_* [°C]** | | | | **Mean *T*_m_** [°C] | | **SD**  [°C] | | **Δ*T*_m_** | |  |
| --- | --- | --- | --- | --- | --- | --- | --- | --- | --- | --- | --- | --- |
|  |  | ramp 2/3/4/5 | | | |  |  |  |  | [°C] | |  |
| R1 | pCGUGCUUA**G**CAUGUACGA | 59.8 | 59.6 | 59.8 | 59.7 | | 59.7 | | 0.1 | | - | |
|  |  |  |  |  |  | |  | |  | |  | |
| R2 | pCGUGCUUA**^tz^G**CAUGUACGA | 59.6 | 59.2 | 59.1 | 60.0 | | 59.4 | | 0.4 | | -0.3 | |
|  |  |  |  |  |  | |  | |  | |  | |
| R1  R4 | pCGUGCUUA**G**CAUGUACGA  GCACGAAU**C**GUACAUGCU | 71.5 | 72.1 | 71.8 | 71.9 | | 71.8 | | 0.3 | | - | |
|  |  |  |  |  |  | |  | |  | |  | |
| R2  R4 | pCGUGCUUA**^tz^G**CAUGUACGA  GCACGAAU **C**GUACAUGCU | 72.2 | 72.8 | 72.4 | 72.6 | | 72.5 | | 0.3 | | +0.7 | |
|  |  |  |  |  |  | |  | |  | |  | |
| R1  R3 | pCGUGCUUA**G**CAUGUACGA  GCACGAAU**A**GUACAUGCU | 60.8 | 60.7 | 60.6 | 60.5 | | 60.7 | | 0.1 | | -11.4 | |
|  |  |  |  |  |  | |  | |  | |  | |
| R2  R3 | pCGUGCUUA**^tz^G**CAUGUACGA  GCACGAAU **A**GUACAUGCU | 60.9 | 61.4 | 61.2 | 61.4 | | 61.2 | | 0.2 | | -10.5 | |
|  |  |  |  |  |  | |  | |  | |  | |
| R1  R5 | pCGUGCUUA**G**CAUGUACGA  GCACGAAU**G**GUACAUGCU | 62.7 | 62.6 | 62.5 | 62.4 | | 62.6 | | 0.1 | | -9.2 | |
|  |  |  |  |  |  | |  | |  | |  | |
| R2  R5 | pCGUGCUUA**^tz^G**CAUGUACGA  GCACGAAU **G**GUACAUGCU | 63.7 | 63.3 | 63.4 | 63.2 | | 63.4 | | 0.2 | | -8.4 | |
|  |  |  |  |  |  | |  | |  | |  | |
| R1  R6 | pCGUGCUUA**G**CAUGUACGA  GCACGAAU**U**GUACAUGCU | 64.9 | 65.1 | 64.9 | 64.8 | | 64.9 | | 0.1 | | -6.9 | |
|  |  |  |  |  |  | |  | |  | |  | |
| R2  R6 | pCGUGCUUA**^tz^G**CAUGUACGA  GCACGAAU **U**GUACAUGCU | 66.0 | 65.9 | 65.8 | 65.8 | | 65.9 | | 0.1 | | -5.9 | |
|  |  |  |  |  |  | |  | |  | |  | |
| R1  R7 | pCGUGCUUA**G**CAUGUACGA  GCACGAAU**I**GUACAUGCU | 61.2 | 61.3 | 61.0 | 61.1 | | 61.2 | | 0.2 | | -10.6 | |
|  |  |  |  |  |  | |  | |  | |  | |
| R2  R7 | pCGUGCUUA**^tz^G**CAUGUACGA  GCACGAAU **I**GUACAUGCU | 63.0 | 63.6 | 63.2 | 63.5 | | 63.3 | | 0.3 | | -8.5 | |
|  |  |  |  |  |  | |  | |  | |  | |
| R1  R8 | pCGUGCUUA**G**CAUGUACGA  GCACGAAU**C_3_**GUACAUGCU | 56.0 | 55.7 | 55.8 | 55.5 | | 55.8 | | 0.2 | | -16.0 | |
|  |  |  |  |  |  | |  | |  | |  | |
| R2  R8 | pCGUGCUUA**^tz^G**CAUGUACGA  GCACGAAU**(CH_2_)_3_**GUACAUGCU | 57.8 | 57.7 | 57.7 | 57.5 | | 57.7 | | 0.1 | | -14.1 | |

**Table S3:** Fitting results of fluorescence lifetime measurements and quantum yields for ^tz^G-modified RNA obtained by relative quantum yield determination to ^tz^G_nuc_.

| **No.** | **5'-NNN-3'**  **3'-NNN-5'** | ***λ*_ex_** [nm]  ***λ*_em_** [nm] | ***A*_1_** | ***τ*_1_**  [ns] | ***A*_2_** | ***τ*_2_**  [ns] | **<*τ*>**  [ns] |  | **QY** |
| --- | --- | --- | --- | --- | --- | --- | --- | --- | --- |
| ^tz^G_nuc_ | - | 318  460 | 0.15 | 0.68 | 0.85 | 9.37 | 8.07 |  | 0.25^[2]^ |
|  |  |  |  |  |  |  |  |  |  |
| R2 | pCGUGCUUA**^tz^G**CAUGUACGA | 318  453 | 0.43 | 3.64 | 0.57 | 13.8 | 9.43 |  | 0.27 |
|  |  |  |  |  |  |  |  |  |  |
| R2  R4 | pCGUGCUUA**^tz^G**CAUGUACGA  GCACGAAU **C**GUACAUGCU | 318  449 | 0.19 | 1.56 | 0.81 | 16.1 | 13.3 |  | 0.39 |
|  |  |  |  |  |  |  |  |  |  |
| R2  R3 | pCGUGCUUA**^tz^G**CAUGUACGA  GCACGAAU **A**GUACAUGCU | 318  448 | 0.31 | 3.31 | 0.69 | 11.0 | 8.62 |  | 0.23 |
|  |  |  |  |  |  |  |  |  |  |
| R2  R5 | pCGUGCUUA**^tz^G**CAUGUACGA  GCACGAAU **G**GUACAUGCU | 318  453 | 0.83 | 3.00 | 0.17 | 9.98 | 4.19 |  | 0.08 |
|  |  |  |  |  |  |  |  |  |  |
| R2  R6 | pCGUGCUUA**^tz^G**CAUGUACGA  GCACGAAU **U**GUACAUGCU | 318  451 | 0.15 | 3.30 | 0.85 | 18.5 | 16.2 |  | 0.42 |
|  |  |  |  |  |  |  |  |  |  |
| R2  R7 | pCGUGCUUA**^tz^G**CAUGUACGA  GCACGAAU **I**GUACAUGCU | 318  453 | 0.40 | 2.98 | 0.60 | 20.7 | 13.6 |  | n.d. |
|  |  |  |  |  |  |  |  |  |  |
| R2  R8 | pCGUGCUUA**^tz^G**CAUGUACGA  GCACGAAU**(CH_2_)_3_**GUACAUGCU | 318  449 | 0.39 | 4.18 | 0.61 | 11.1 | 8.40 |  | 0.20 |

**Table S4:** HOMO and LUMO energies *E*_HOMO_ and *E*_LUMO_ with the corresponding band gap Δ*E* for each canonical RNA nucleobase, ^th^G and ^tz^G. Geometry optimization and calculations were done at ωB97X-D3/TZVP level of theory.

| **No.** | ***E*_HOMO_ (eV)** | ***E*_LUMO_ (eV)** |  | **Δ*E*(eV)** | **Exp. VIP (eV)^[10]^** |
| --- | --- | --- | --- | --- | --- |
| A | -8.3896 | 1.3481 |  | 9.7377 | 8.3–8.5 |
|  |  |  |  |  |  |
| C | -8.7995 | 1.0531 |  | 9.8526 | 8.8–9.0 |
|  |  |  |  |  |  |
| G | -8.1077 | 1.7995 |  | 9.9072 | 8.0–8.3 |
|  |  |  |  |  |  |
| U | -9.4920 | 0.6988 |  | 10.1908 | 9.4–9.6 |
|  |  |  |  |  |  |
| ^th^G | -8.1465 | 0.5586 |  | 8.7051 | n.d. |
|  |  |  |  |  |  |
| ^tz^G | -8.5107 | 0.1253 |  | 8.6360 | n.d. |

**Table S5:** Overview of observed rate constants *k*_obs_ for the reaction of the 8-17 deoxyribozyme variant D1 with unmodified (R1) and modified (R2) substrate and 8-17 variants D2 and D3 with unmodified (R9) and modified (R10) substrate. Shown are *k*_obs_ values for each experimental replicate (rep.), the respective mean and standard deviation (SD) as well as the relative rate constant *k*_rel_ as the quotient of *k*_obs_(unmodified) and *k*_obs_(modified). Kinetic assays were performed under single turnover conditions with 1 µm substrate RNA and 10 µm deoxyribozyme.

| **No.** | **description** | ***k*_obs_ [min^-1^]** | | | **Mean *k*_obs_ [min^-1^]** | **SD** | ***k*_rel_** |
| --- | --- | --- | --- | --- | --- | --- | --- |
|  |  | **rep. 1** | **rep. 2** | **rep. 3** |  |  |  |
| R1/D1 | 20 mm MgCl_2_, pH7.5, 37 °C | 0.0201 | 0.0161 | 0.0195 | 0.0186 | 0.0022 | 4.9 |
| R2/D1 |  | 0.0035 | 0.0042 | 0.0037 | 0.0038 | 0.0004 |  |
|  |  |  |  |  |  |  |  |
| R1/D1 | 10 mm MgCl_2_, 10 mm MnCl_2_, pH7.5, 25 °C | 0.1163  0.0190 | 0.1245  0.0169 | 0.1183  0.0211 | 0.1197 | 0.0043 | 6.3 |
| R2/D1 |  |  |  |  | 0.0190 | 0.0021 |  |
|  |  |  |  |  |  |  |  |
| R1/D1  R2/D1 | 10 mm MgCl_2_, 10 mm MnCl_2_, pH7.5, 37 °C | 0.1374  0.0249 | 0.1737  0.0251 | 0.1466  0.0243 | 0.1526  0.0248 | 0.0189  0.0004 | 6.2 |
|  |  |  |  |  |  |  |  |
| R1/D1  R2/D1 | 10 mm MgCl_2_, 10 mm MnCl_2_, pH8.0, 37 °C | 0.7557  0.0564 | 0.7660  0.0518 | 0.7481  0.0510 | 0.7566  0.0531 | 0.0090  0.0029 | 14.2 |
|  |  |  |  |  |  |  |  |
| R9/D2  R10/D2 | 20 mm MgCl_2_, pH7.5, 25 °C | 0.0187  0.0025 | 0.0198  0.0037 | 0.0161  0.0040 | 0.0182  0.0034 | 0.0019  0.0008 | 5.4 |
|  |  |  |  |  |  |  |  |
| R9/D2 | 10 mm MgCl_2_, 10 mm MnCl_2_, pH7.5, 25 °C | 1.0464  0.0623 | 1.0090  0.0597 | 1.1223  0.0571 | 1.0592 | 0.0577 | 17.7 |
| R10/D2 |  |  |  |  | 0.0597 | 0.0026 |  |
|  |  |  |  |  |  |  |  |
| R9/D2  R10/D2 | 10 mm MgCl_2_, 10 mm MnCl_2_, pH7.5, 37 °C | 1.2545  0.0419 | 1.2204  0.0395 | 1.2937  0.0366 | 1.2562  0.0393 | 0.0367  0.0027 | 32.0 |
|  |  |  |  |  |  |  |  |
| R9/D3  R10/D3 | 20 mm MgCl_2_, pH7.5, 25 °C | 0.0351  0.0004 | 0.0384  0.0013 | 0.0378  0.0017 | 0.0371  0.0011 | 0.0018  0.0006 | 33.7 |
|  |  |  |  |  |  |  |  |
| R9/D3 | 10 mm MgCl_2_, 10 mm MnCl_2_, pH7.5, 25 °C | 0.0645  0.0384 | 0.0701  0.0444 | 0.0687  0.0403 | 0.0678 | 0.0029 | 1.7 |
| R10/D3 |  |  |  |  | 0.0410 | 0.0031 |  |
|  |  |  |  |  |  |  |  |
| R9/D3  R10/D3 | 10 mm MgCl_2_, 10 mm MnCl_2_, pH7.5, 37 °C | 0.1472  0.0091 | 0.1209  0.0090 | 0.1357  0.0115 | 0.1346  0.0099 | 0.0132  0.0014 | 13.6 |

# Supporting figures

**Figure S1** Secondary structures of human tRNA-Ala-AGC (a) and mitochondrial tRNA-Trp (b). The fragments used in this study are colored red. The guanosine which was replaced by ^tz^G is circled.

**Figure S2** HPLC traces for crude oligonucleotides R2 (a) and R10 (b) measured at 260 nm. Anion exchange HPLC, Dionex, DNAPac PA200, 2x250 mm, 60 °C.

**Figure S3** HPLC traces for pure oligonucleotides measured at 260 nm: R1 (a), R2 (b), R3 (c), R4 (d), R5 (e), R6 (f), R7 (g), R8 (h), R9 (i), R10 (j), D1 (k), D2 (l), D3 (m) and MTR1 (n). Anion exchange HPLC, Dionex, DNAPac PA200, 2x250 mm, 60 °C.

**Figure S4** Native (non-urea containing) PAGE images of annealed RNA samples with either R1 (a) or R2 (b) stained with SYBR gold. Hairpin structures migrate lower compared to duplex structures. Conditions: 1 pmol RNA, 15% native PAGE, 100 V, 1 h run.

**Figure S5** Melting curves for 2 µm samples of hairpin and duplex structures measured at 260 nm. Conditions: 2  µm RNA in PBS buffer (100 mm NaCl, 10 mm phosphate, pH7.4), 0.5 °C/min heating rate.

**Figure S6** Relative quantum yield determination for ^tz^G-modified structures. Shown are the absorption spectra (left), the fluorescence emission spectra (middle) and a plot of the integrated fluorescence emission F versus the absorption factor a with a linear fit (right). Conditions: 2, 5 and 7 μm nucleoside or RNA in 1x PBS (100 mm NaCl, 10 mm phosphate, pH7.4).

**Figure S7** HOMO and LUMO energies of canonical nucleobases, ^th^G and ^tz^G and frontier molecular orbital distributions calculated at ωB97X-D3/TZVP level of theory.

**Figure S8** Fluorescence lifetime measured by TCSPC. Decays for RNA samples are shown in red, the instrument response function is shown in black and the biexponential fit is shown in blue. The average lifetime <τ> is given for each sample. Conditions: 1 μm nucleoside or RNA in 1x PBS (100 mm NaCl, 10 mm phosphate, pH7.4).

**Figure S9** 8-17 Deoxyribozyme-substrate constructs R1 or R2 with D1 (a), R9 or R10 with D2 (b) or D3 (c) with the cleavage site indicated by an arrow and the G that was replaced by ^tz^G shown in bold.

**Figure S10** Denaturing PAGE analyses for cleavage assays of deoxyribozymes D1 to D3 with fluorescein-labelled unmodified RNA substrates R1 and R9 and modified RNA substrates R2 and R10 at different temperatures and in the presence of different concentrations of bivalent cations. Kinetic assays were done under single-turnover conditions with 1 µm of RNA and 10 µm of DNAzyme using the conditions indicated above each panel.

**Figure S11** Plot of cleaved RNA fraction from reaction of D1 to D3 with unmodified RNA substrates R1 or R9 and modified RNA substrates R2 and R10. Shown is the mean of three independent replicates with standard deviation of the mean shown as error bars. Data were fitted according to a first-order kinetics equation Y = Y_max_ * (1 − e^−^*^k^*^obs*^*^t^*).

**Figure S12** Reverse-phase HPLC chromatogram of the cleavage reaction of ^tz^G-containing substrate RNA R2 with the 8-17 deoxyribozyme variant D1 after overnight incubation at 37 °C with (conditions: 1 µm RNA, 10 µm DNA, 50 mm Tris-HCl, 150 mm NaCl, pH8.0, 10 mm MgCl_2_, 10 mm MnCl_2_). Chromatograms were recorded at an absorption wavelength of 260 nm and a fluorescence emission wavelength of 349 nm. The area in the red box is shown enlarged on the right side of the figure. Conditions: buffer A (100 mm TEAA pH7.0), buffer B (100 mm TEAA pH7.0 in 25% MeCN), 60 °C,15 to 60% buffer B over 26 minutes.

**Figure S13** Steady-state fluorescence spectra for R2 in 1x PBS (100 mm NaCl, 10 mm phosphate, pH7.0), 1x kinetic assay buffer (KAB, 50 mm Tris-HCl, 150 mm NaCl, pH8.0), 1x kinetic assay buffer with 20 mm MgCl_2_ and 1x kinetic assay buffer with 10 mm MgCl_2_ and 10 mm MnCl_2_ (a) and in the presence of different divalent cations (b).

**Figure S14** Steady-state fluorescence spectra for a complex of D1 and R2 after incubation at 37 °C for 5 h in the presence of either kinetic assay buffer or stop solution (contains 80% formamide). Reaction samples contained 1 µm RNA, 10 µm DNA as well as 10 mm MgCl_2_ and 10 mm MnCl_2_ in addition and samples were measured at either 25 °C (a) or 37 °C (b).

**Figure S15** (a) MTR1-substrate construct with the alkylation site indicated by an arrow, the modified G shown in bold and nucleobases in contact with ^tz^G shown in red. (b) Simulated and deconvoluted HR-ESI mass spectrum for R10 methylated at A9 with MTR1. (c) Analytical ion exchange HPLC traces for incubation of R9 or R10 with MTR1 in the presence or absence of m^6^G. The signal for the methylated substrate is shifted to lower retention times due to the positively charged m^1^A in the methylated substrate. (d) Fluorescence emission spectra for reaction mixture of R10 and MTR1 after overnight incubation without guanosine substrate (black) and in the presence of m^6^G (blue) or Bn^6^G (red). Conditions for methylation reaction: 35 µm RNA R9 or R10, 45 µm MTR1, 200 µm alkyl^6^G in buffer (50 mm BisTris, 120 mm KCl, 5 mm NaCl, pH6.0), incubation for 16 h at 25 °C.

# NMR spectra

**Figure S16** ^1^H-NMR spectrum (400 MHz, DMSO-*d*_6_) of compound **S15**.

**Figure S17** ^13^C-NMR spectrum (100 MHz, DMSO-*d*_6_) of compound **S15**.

**Figure S18** ^1^H-NMR spectrum (400 MHz, CDCl_3_) of compound **S16**.

**Figure S19** ^13^C-NMR spectrum (100 MHz, CDCl_3_) of compound **S16**.

**Figure S20** ^1^H-NMR spectrum (400 MHz, CDCl_3_) of compound **S18**.

**Figure S21** ^13^C-NMR spectrum (100 MHz, CDCl_3_) of compound **S18**.

**Figure S22** ^1^H-^1^H COSY NMR spectrum (CDCl_3_) of compound **S18**. The important H-H couplings are marked; the coupling between 3'-H and 3'-OH proton confirms the identity of the desired 2'-TBDMS isomer.

**Figure S23** ^1^H-NMR spectrum (400 MHz, CDCl_3_) of compound **S19**.

**Figure S24** ^13^C-NMR spectrum (100 MHz, CDCl_3_) of compound **S19**.

**Figure S25** ^31^P-NMR spectrum (162 MHz, CDCl_3_) of compound **S19**.

# References

[1]	K. Wawrzyniak-Turek, C. Höbartner, "Enzymatic combinatorial nucleoside deletion scanning mutagenesis of functional RNA" *Chem Commun (Camb)* **50**, (2014): 10937.

[2]	A. R. Rovira, A. Fin, Y. Tor, "Expanding a fluorescent RNA alphabet: synthesis, photophysics and utility of isothiazole-derived purine nucleoside surrogates" *Chem Sci* **8**, (2017): 2983.

[3]	M. Ghazzali, S. A. N. Khattab, Y. A. Elnakady, F. A. Al-Mekhlafi, K. Al-Farhad, A. El-Faham, "Synthesis, structure, theoretical calculations and biological activity of sulfonate active ester new derivatives" *J. Mol. Struct.* **1046**, (2013): 147.

[4]	C. Steinmetzger, C. Bäuerlein, C. Höbartner, "Supramolecular Fluorescence Resonance Energy Transfer in Nucleobase-Modified Fluorogenic RNA Aptamers" *Angew Chem Int Ed Engl* **59**, (2020): 6760.

[5]	F. Neese, F. Wennmohs, U. Becker, C. Riplinger, "The ORCA quantum chemistry program package" *J Chem Phys* **152**, (2020): 224108.

[6]	F. Neese, "Software Update: The ORCA Program System-Version 6.0" *Wires Comput Mol Sci* **15**, (2025).

[7]	Y. S. Lin, G. D. Li, S. P. Mao, J. D. Chai, "Long-Range Corrected Hybrid Density Functionals with Improved Dispersion Corrections" *J Chem Theory Comput* **9**, (2013): 263.

[8]	F. Weigend, R. Ahlrichs, "Balanced basis sets of split valence, triple zeta valence and quadruple zeta valence quality for H to Rn: Design and assessment of accuracy" *Phys Chem Chem Phys* **7**, (2005): 3297.

[9]	D. Rappoport, F. Furche, "Property-optimized gaussian basis sets for molecular response calculations" *J Chem Phys* **133**, (2010): 134105.

[10]	D. Roca-Sanjuan, M. Rubio, M. Merchan, L. Serrano-Andres, "Ab initio determination of the ionization potentials of DNA and RNA nucleobases" *J Chem Phys* **125**, (2006): 084302.
